# The causal relationship model of factors influencing COVID-19 preventive behaviors during the post-pandemic era and implications for health prevention strategies: a case of Bangkok City, Thailand

**DOI:** 10.1186/s12879-024-09818-8

**Published:** 2024-08-29

**Authors:** Piyapong Janmaimool, Jaruwan Chontanawat, Siriphan Nunsunanon, Surapong Chudech

**Affiliations:** 1 ASEAN Institute for Health Development, Mahidol University, Salaya, Phuttamonthon 4 Road, 999, 73170 Nakorn Pathom Province Thailand; 2 The Joint Graduate School of Energy and Environment (JGSEE), King Mongkut’s University of Technology Thonburi, 126 Pracha-Uthit Rd., Thungkru, Bangkok, 10140 Thailand; 3 Department of Social Sciences and Humanities, School of Liberal Arts, King Mongkut’s University of Technology Thonburi, 126 Pracha-Uthit Rd., Thungkru, Bangkok, 10140 Thailand

**Keywords:** Risk perception, Health communication, Health protective behaviours, Risk characteristics, SARS-CoV-2, COVID-19

## Abstract

**Background:**

Though, many countries are currently in the COVID post-pandemic era, people’s health protective behaviours are still essential to protect their health and well-being. This study aims to evaluate people’s understanding and perceptions of COVID-19 risk characteristics (i.e. threat occurrence, threat severity, perceived susceptibility and exposure), the health risk perception towards COVID-19, and health protective behaviours. The study also aims to estimate the associations among these factors by the analysis of structural equation modelling (SEM).

**Methods:**

From 15 October to 9 November 2022, questionnaire surveys were administrated to 521 people living in Bangkok of Thailand by using the convenience sampling technique. The analyses were carried out in three phases including descriptive statistical analyses, a measurement model assessment using a confirmatory factor analysis (CFA), and structural equation modelling (SEM) analysis.

**Results:**

The results of descriptive analyses demonstrated that the majority of respondents, 39.9%, had the age between 20 and 30 years old, and 61.4% of them were female. Approximately 52.1% of them had a bachelor’s degree. Upon analysing individuals’ understanding and perceptions of all risk characteristics, individuals’ understanding of COVID-19 severity did not statistically affect health risk perception towards COVID-19, whereas perceived exposure had the strongest effect and in turn influenced health protective behaviours. Perceived susceptibility and understanding of the threat occurrence also significantly affected health risk perception, and indirectly affected health protective behaviours.

**Conclusions:**

This study implies that though the potential health impact of COVID-19 is perceived as less severe, people can still construct a perception of its risk particularly based on their perceived exposure and susceptibility. Thus, communicating people about exposure conditions and susceptibility can greatly contribute to people’ construction of risk perception towards COVID-19 which subsequently leads to the decision to perform health protective behaviours.

## Highlights

This study investigated whether people still construct a perception of COVID-19 related risk during the post-pandemic era.

Though the potential health impact of COVID-19 is perceived as less severe, people still constructed a perception of its risk particularly based on their perceived exposure and susceptibility.

Risk perceptions of COVID-19 during the post-pandemic era could explain only 10–23% of variances in heath protective behaviours.

Upon analysing individuals’ perceptions of all risk characteristics, perceived severity of COVID-19 did not statistically affect risk perception, whereas perceived exposure had the strongest effect.

Perceived susceptibility and occurrence of COVID-19 spreading also significantly affected risk perceptions, and indirectly affected health protective behaviours.

Thus, communicating people about exposure conditions and susceptibility is effective to promote heath protective bahaviours during the post-pandemic era.

## Introduction

Since the first emergence of SARS-CoV-2 (COVID-19) pandemic in early December 2019, massive efforts to control and manage the virus transmissions have been implemented. Healthcare workers as well as individuals have been encouraged to perform health protective behaviours against the virus. Though, the COVID-19 situations have been improving in several countries, the COVID-19 is still a public health threat which requires individuals’ health protective actions and effective health systems. The health impact of COVID-19 can be very devastating as the virus potentially causes severe respiratory illness [1, 2], and subsequently leads to death [3, 4]. The physical symptoms of COVID-19 are such as fever, cough, difficulty breathing, sore throat, headache, loss of smell or taste, and conjunctivitis [5, 6]. Several studies reported sever health impacts of COVID-19. Jiménez-Zarazúa et al. [7] reported that many COVID-19 patients developed acute respiratory distress syndrome, a pathology which can potentially cause chronic lung damage. Pereckaitė et al. [6] added that COVID-19 patients could develop organ damage including myocarditis and pericarditis.

By mid-October 2022, it was reported by World Health Organization (WHO) that approximately 621 million people had contracted the virus, and 6.5 million people died [8]. Since, the COVID-19 pandemic had been declared by WHO as a global health emergency in March 2020, the WHO and the governments of each country recommended both medical staff and the general population use COVID-19 Personal Protective Equipment (PPE), such as medical and non-medical face masks (e.g. self-made masks of cloth, cotton or other textiles), face shields, aprons and gloves [9]. The current COVID-19 situation has become better than the situation in the last three years, and the WHO decided to declare an end of global emergency status for COVID-19 in May 2023 [10]. However, health impacts of COVID-19 still exist. Many people, particularly vulnerable groups (e.g., elderly people with age over 60 years, people with underlying health conditions such as diabetes, heart disease, and respiratory illnesses, and pregnant women) [11], can be killed by this virus, and there is a high chance that new variants will occur and consequently cause new cases and deaths. WHO [12] states that it is still necessary to continue protecting people, particularly the most vulnerable group against the virus. WHO also recommends that people should continue to take the preventive actions needed to protect their health. The continuous use of personal protective equipment (PPE) by healthcare workers and individuals is recommended by WHO [8]. Essential PPE includes gloves, medical masks, goggles or a face shield, and gowns, as well as for specific procedures, respirators, and aprons.

Since the first occurrence of COVID-19 in Thailand, the Thai population has been encouraged to perform health protective behaviours. Many Thai population were active to wear face masks during the pandemic era (2020–2021) [13]. However, the Thai government by the Ministry of Public Health has announced that Thailand entered to the post-pandemic era since June 2022, and the cancellation of the state of emergency declaration was also announced in September 2022, due to a decrease in death rate, and a high percentage of vaccination coverage [14]. As of 15th November 2022, 54 provinces out of 77 provinces achieved 2-dose vaccination coverage of more than 70% [14], and the Case Fatality Rate (CFR) was reduced from 1.89% in January 2022 to 0.01% at the end of October 2022. Though, COVID-19 pandemic is improving in Thailand, it is still important to maintain health protective behaviours.

It becomes challenging to encourage people to continue preforming health protective behaviours against COVID-19, as they have become familiar with the situation, and have tended to forget to perform such protective behaviours against COVID-19. The focus of this research is thus examining factors influencing people’s health protective behaviours against COVID-19. Several scholars have stated that individuals’ health protective behaviours are greatly influenced by their risk perception of the pandemic, thus leading to these behaviours. For instance, Wismans et al. [15] revealed that the perceived health risk of COVID-19 positively affects face mask use. According to the Health Belief Model (HBM) [16] and Protection Motivation Theory (PMT) [17], health protective behaviours are influenced by individuals’ risk perceptions (described in PMT as individuals’ threat appraisal). Understanding risk perception, its determinants and its association with health protective behaviours can reveal how to develop communication strategies which can enhance people’s motivation to perform the protective behaviours. Bruine de Bruin and Bennett [18] confirmed that individuals with a greater risk perception are more likely to perform health protective behaviours. Similarly, many studies conducted during the COVID-19 outbreak have confirmed that risk perception is related to the implementation of COVID-19 prevention behaviours [19–21]. However, what should be elaborated are determinants of risk perception which can be diverse. Understanding determinants of risk perception can provide basic understanding on how to maintain people’s constructed risks associated with COVID-19 which consequently influence their health protective behaviours.

In this way, how people judge and perceive the risks associated with COVID-19 can affect their performance of health protective behaviours. Based on the psychometric paradigm developed by Slovic [22], risk perception can be constructed based on individuals’ rational thinking process by considering a combination of (perceived) risk characteristics, such as perceived severity of the risk, perceived exposure to the risk, controllability, familiarity and observability. Fischhoff et al.’s [23] research in the modern day implies that individuals’ perceived risks of the COVID-19 can be amplified or attenuated due to differences in individuals’ understanding or judgement of risk characteristics. Regarding risk perception towards COVID-19 pandemic, Lohiniva at al. [24] indicated that individuals’ interpretation, comprehension, understanding and perceptions of the virus characteristics could have a significant impact on individuals’ health risk perception. The virus characteristics are such as the scope of pandemic, the severity of the symptoms caused by infection, the risk of virus transmission, virus exposure environments, and vulnerable health conditions. Cardona et al. [25] classified risk characteristics into three aspects.

The first one is hazard, which refers to the possible, future occurrence of undesirable events that may have adverse effects on vulnerable and exposed elements [26, 27]. The second aspect is exposure, or the inventory of elements in an area in which hazardous events may occur [28]. In the event that people are not living in potentially dangerous settings, no problem of disaster risk would exist. The last aspect is vulnerability, which refers to the propensity of exposed elements such as human beings, their livelihoods and assets suffering adverse effects when impacted by hazard events [27]. Vulnerability relates to susceptibilities, fragilities, weaknesses, deficiencies or insufficient capacities that cause adverse effects for exposed elements. COVID-19 can be considered a type of disaster event. Therefore, the ways individuals understand and perceive characteristics of this pandemic risk, including hazard, exposure and vulnerability, may shape their health risk perceptions, which consequently leads to participation in health protective behaviours.

Understanding what is driving the health risk perception can allow risk communicators to communicate with the public about risks, subsequently promote behavioural change [24]. Based on the psychometric paradigm [22], this study assumes that individuals use their rational thinking to judge risk of COVID-19, and thus are motivated to perform health protective behaviours. Through rational thinking, individuals’ health risk perceptions can be determined by how they understand and judge risk characteristics [22, 23]. This study divides risk characteristics into three aspects: perceived exposure, vulnerability and hazard, which includes threat occurrence and severity [25]. Differences in individual judgements of these risk characteristics may affect health risk perception, thus leading to a difference in health protective behaviours. Currently, the COVID-19 outbreak situation is changing over time due to the consecutive emergence of new coronavirus variants and vaccination types. This change may influence the way people construct risks and health responses.

Accordingly, this study aims to examine people’s risk perception towards COVID-19 during the post-pandemic era, and examine how the risk perception towards COVID-19 have been influenced by the understanding and perceptions of risk characteristics (i.e. threat occurrence, threat severity, individual susceptibility and exposure). Finally, the effect of risk perception on health protective behaviours will be examined. The casual relationship of factors influencing health protective behaviours against COVID-19 will be evaluated by the analysis of structural equation modelling (SEM). Bangkok city of Thailand was selected as a case study because it is a highly populated area and individuals’ heath protective behaviours against COVID-19 should be strongly promoted. By understanding the association among factors influencing individuals’ health protective behaviours could help provide significant implications for the development of communication strategies to promote protective behaviours against COVID-19 during the post-pandemic era.

## Literature review and hypotheses

### Health protective behaviours

Several studies revealed effectiveness of health protective behaviours in reducing risks of infection [29–32]. For instance, the study of Lio et l. [30] revealed that outdoor mask wearing could reduce COVID-19 risk by 69.3% after adjusting for other confounders such as contact history, hygiene practice, and being in crowded activities. The study of Hajmohammadi et al. [31] reported that the application of PPE or facial mask use was significantly associated with a decrease in risk of COVID-19 infections. In Thailand, people are especially encouraged to participate in COVID-19 self-preventive measures. First, since ATKs were approved for home use by the Ministry of Public Health Thailand’s Food and Drug Administration, people have been encouraged to use them to test for COVID-19 infection when they have suspicious symptoms, or when they must be in a crowded and inadequately ventilated space. Though they have low sensitivity, immunochromatographic assay rapid antigen test (RAT) and ATK kits are affordable and accessible to the general public. RAT kits require minimal training and equipment, and are very useful for the identification of infected people [33]. The purpose of RAT kits is to detect the nucleocapsid protein of COVID-19 in nasal swab specimens [34]. RATs can detect the presence of a specific viral antigen, which implies COVID-19 infection. Currently authorized methods may include point-of-care tests and at-home self-tests, and are applicable to people of any age [35].

Second, people in Thailand are encouraged to use face masks. According to the WHO’s COVID-19 advice for the public [36], people are encouraged to wear a mask as a normal part of being around others if COVID-19 is spreading in their community. Several studies have revealed the effectiveness of wearing a face mask in preventing the spread of COVID-19 [37]. Chua et al. [38] and Pullangott & Kannan [39] demonstrated that droplets containing the virus can be filtered by face masks. Face masks have been utilized as a public and personal health control measure, and have been widely implemented to control the spread of COVID-19 [40]. Moreover, several studies have shown that wearing two masks creates more filtration efficiency than just one, and can substantially reduce individuals’ exposure to the virus [41, 42]. In Thailand, according to an order published in the *Royal Gazette* in June 2021, people were required to wear a face mask in public places [43]. However, on 23 June 2022, the wearing of face masks became voluntary, though many parties still encourage people to do so [44].

### Risk perception towards COVID-19 and health protective behaviours

Risk perception refers to a subjective assessment of a potential threat to individuals’ lives or psychological well-being [41]. Lohiniva at al. [24] explain risk perception as one’s subjective assessment of the actual or potential threat to one’s life or one’s psychological well-being. Slovic [45] defines risk perception as the assessment of the severity and probability of negative outcomes. Regarding risk perception towards COVID-19, risk perception consists of two aspects including the probability of being infected by the virus (i.e., infection probability) and the perceived severity of the symptoms after actual infection (i.e., outcome severity) [46, 47]. For instance, Adachi et al. [48] measured risk perception of COVID-19 based on individuals’ perceived possibility of being infected with the COVID-19 and severity of severe illness caused by the infection. Risk perception can be estimated with respect to one’s personal situation or general population at large.

The Protection Motivation Theory (PMT) [17] explains risk perception (threat appraisal) as individuals’ estimation of the level of threat to themselves and their valued things which relatively influence behavioural responses against their facing threat. Based on PMT, risk perception contains two aspects: individuals’ perceived severity of the threat and their perceived probability of facing adverse impacts from the threat. PMT has been widely and successfully applied in the context of health threats to explain how people’s feelings of fear affect their health response or health behaviours [49]. To measure health risk perception based on PMT, researchers examine individuals’ beliefs in the severity of the threat to their valued things (perceived severity) and their estimation of the chance of being affected by the health risk (perceived vulnerability) [50, 51]. Like PMT, Becker’s [52] Health Belief Model (HBM) also explains risk perception, particularly in the context of health, as individuals’ feelings of the seriousness or harmfulness of contracting a disease, and individuals’ perceived possibility of contracting an illness or disease. Both theories assume that health risk perceptions affect health preventive behaviours.

These theories have been applied in many studies to explore determinants of health behaviours. For instance, Becker [52] utilized the HBM to find that health behaviours are driven by individuals’ risk perceptions of disease susceptibility and severity. Regarding the 2003 Severe Acute Respiratory Syndrome (SARS) outbreak, many studies revealed that higher perceptions of SARS infection were significantly related to engagement in more preventive behaviours and compliance with disease control strategies [49, 53]. Further, Siegrist and Bearth [54] concluded that perceived threat to individuals’ environment influences their compliance with protective measures. With COVID-19, several studies have also demonstrated COVID-19’s perceived social risk to be associated with engagement in protective measures [55, 56]. Asri et al. [57] revealed that among younger age groups, the perceived threat of COVID-19 to other people beyond themselves was more influential in affecting their decision to wear masks; in contrast, older people were motivated to wear a mask based on their perceived threat to themselves. Wise et al. [58] found that individuals’ health risk perceptions affect their compliance with COVID-19 measures. In general, both self- and other-related risk perceptions are assumed to have a positive effect on individuals’ decision to wear a mask.

The Ministry of Public Health of Thailand has promoted three types of COVID-19 self-preventive measures, which are considered effective to reduce risks of COVID-19 infections [59]. These measures are wearing a face mask outside the home or double face masks in highly crowded or poorly ventilated places, and using RAT kits for COVID-19 detection, as widely encouraged by many parties (e.g. educational institutes, companies and government offices). This study accordingly assumes that individuals’ practice of COVID-19 preventive measures can be predicted by health risk perception. Beyond exploring the effect of risk perception on decisions to perform health preventive behaviours, as widely reported in many relevant studies [55, 59], this study intends to reveal the effect of health risk perception on the intensity of health preventive practices, as reflected by the degree of PPE use. Namely, when individuals perceive a high health risk, they tend to perform intensive practice of health preventive behaviours, such as wearing a face mask and using ATKs for COVID-19 detection.

### Understanding and perceptions of risk characteristics as factors influencing risk perception and Health Protective behaviours

Based on the psychometric paradigm [22], individuals’ risk perception is based on their rational thinking process, itself based on their interpretation, comprehension and understanding of risk characteristics. Cardona et al. [25] classified risk characteristics into three dimensions: hazard, vulnerability and exposure to a harmful event. In the psychometric paradigm, risk perception can be generated from the evaluation of these risk characteristics, which fall under “unknown risk” or “dread risk” [22]. Dread risk refers to individuals’ perception of catastrophic consequences of a threat or harmful event, and perception of their control over exposure to that risk. These perceptions can affect individuals’ feelings of fear and drive their motivation to perform response behaviours. The more fear that individuals construct when being exposed to a risk, the more they tend to perceive the risk as higher [60, 61]. In turn, unknown risk refers to the characteristics of a harmful event, particularly if it is familiar, predictable, observable and understood [62, 63]. Risks can be perceived as high if individuals are not familiar with the harm; risks might also have delayed effects.

For this study, individuals’ understanding, and perceptions of all risk characteristics (hazard, susceptibility and exposure) are deliberatively explored to determine whether and how they affect risk perception. The first risk characteristic is hazard. Each hazard has two particular features that individuals perceive differently according to their understanding, comprehension, and interpretation: threat occurrence and threat severity. With threat occurrence, individuals who recognize the possibility of threat occurrence start assessing the risk that they may face, and consequently construct risk perception. Meanwhile, threat severity refers to the threat’s catastrophic consequence(s). According to the psychometric paradigm [22], individuals who are aware of a threat’s catastrophic consequences are more likely to construct risks. Saito et al. [64] revealed that threat occurrence (e.g. earthquakes) significantly affects risk perception. Regarding COVID-19, Lohiniva et al. [24] found that individuals’ understanding of the nature of the virus, such as its potential health impacts, affect their health risk perception.

The second risk characteristic that can influence risk perception is risk susceptibility – in this case, specifically disease susceptibility. Disease susceptibility refers to conditions in which individuals can be easily affected by a harmful medical event, such as having poor health conditions, a chronic disease or limited capacity to cope with a disease. For instance, Ritz et al. [65] noted that health status, particularly respiratory or allergen-based illnesses, is relatively associated with air pollution perception. McCormack et al. [66] told that obesity makes individuals with chronic obstructive pulmonary disease (COPD) susceptible to indoor particulate matter. Socio-economic characteristics can also be associated with personal susceptibility. For example, younger people construct a lower risk perception of COVID-19 than older people [67], because the young population generally has better health. Ding et al. [68] revealed that female and non-medical students construct a higher level of perceived risk to COVID-19 than male and medical students. Many previous studies revealed that individuals’ perceived susceptibility to disease can increase their health risk perception. For instance, in the context of the COVID-19 pandemic, individuals with different degrees of perceived susceptibility show significant differences in their risk perception of the virus [69]. Makhanova and Shepherd [70] indicated that individuals’ perceived vulnerability to disease contributes to stronger reactions to the COVID-19 threat, including an increased degree of anxiety, demand for behavioural change and higher importance granted to proactive behaviour. The study of Adachi et al. [48] found that perceived poor health conditions were significantly associated with high-risk perception towards COVID-19 infection and illness. Several studies have explored how perceived susceptibility directly affects health protective behaviours as well [71]. For instance, Tang and Wong [72] found that Chinese citizens who constructed a low level of perceived susceptibility to the 2003 SARS epidemic tended to participate less in health protective behaviours, such as wearing face masks and sanitizing hands.

The last type of risk characteristic is exposure. Individuals’ exposure to a threat can determine their perceived risk as feeling exposed to a threat, which in turn can make them aware of the possibility of its negative impacts. Orru et al. [73], for instance, found that individuals’ perceived exposure to PM_10_ determines their health risk perception, which consequently influences health symptoms due to stress and anxiety. Lee et al. [74] revealed that when exposed to an individual sneezing in public, individuals’ perceived risks to potential threats relatively increase. Similarly, Koh et al. [75] found that an increase in health risk perception was determined by individuals’ perceived exposure to the electromagnetic waves emitted from 5G network base stations. In terms of COVID-19 transmission, the virus can travel on droplets that might be larger or smaller than 5 μm. Exhaled droplets over 5 μm will fall to the ground within some distance from the exhaling person [76], whereas droplets smaller than 5 μm (called aerosols) originate directly from exhalation and can stay in the air for a long time [77]. Aerosols can then provide ambient virus exposure. Individuals’ exposure to viral transmission can therefore be attributed to moving between places [78], participating in face-to-face activities [79], being in crowded environments [80] and being in poorly or non-ventilated spaces [80, 81]. Hong et al. [82] notably found that areas with higher population flows have more COVID-19 infection rates. People have also been widely educated about the nature of virus transmission, and can therefore construct a perception of their exposure which might consequently influence their health risk perceptions, and their decision to perform protective behaviours.

**Fig. 1:**
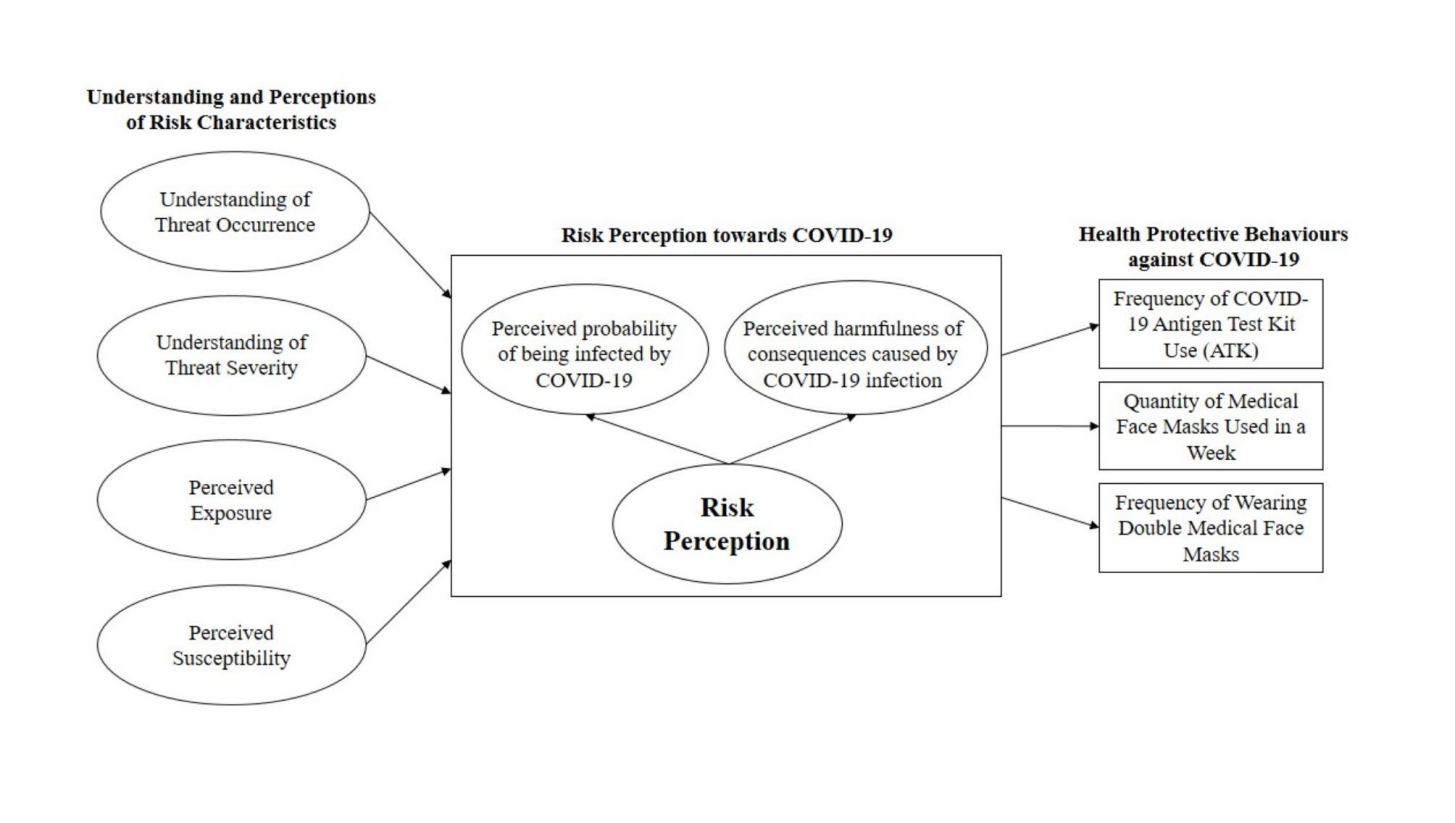
Conceptual framework of this study

Currently, the COVID-19 situation is ever-changing due to the consecutive emergence of new coronavirus variants, vaccination developments and changes in the mitigation and prevention of measures such as lock-downs. This study assumes that these changes affect people’s understating, interpretation and perceptions of risk characteristics related to COVID-19, which in turn, affect their risk perception. As discussed above, risk perception of COVID-19 is the sum of two aspects including individuals’ perceived infection probability and perceived outcome severity or perceived severity of the symptoms after actual infection [46, 47, 83]. Based on theoretical discussion, these two components of risk perception can be evaluated and perceived differently by individuals based on the understanding and perceptions of risk characteristics including (1) understanding of threat occurrence (e.g., virus transmission, locations of virus transmission, and an occurrence of pandemic) (2) understanding of threat severity (e.g., possibility of death, the severity of the symptoms associated with the virus, or possible sever illness), (3) perceived exposure to COVID-19 transmission (e.g., exposure conditions) and (4) perceived susceptibility to COVID-19 (e.g., sensitiveness to the impacts). Once risks of COVID-19 are perceived in some level, it is likely that individuals will perform behavioural responses or recommended health protective behaviours against COVID-19. Risk perceptions can play an important role in driving motivation to perform actions to eliminate risks. In another word, risk perceptions of COVID-19 can provide a legitimate reason for individuals to endorse the significance of recommended health protective behaviours. For instance, the study of Schmitz et al. [84] revealed that individuals with a high perception of severe illness after COVID-19 infection reported higher motivation to uptake vaccination, which in turn affected an effective uptake of the COVID-19 vaccine. Based on this discussion, the following research hypotheses can thus be proposed (see Fig. 1):

#### H1

People’s understanding of threat occurrence can affect health protective behaviours against COVID-19 via their risk perception towards COVID-19.

#### H2

People’s understanding of threat severity can affect health protective behaviours against COVID-19 via their risk perception towards COVID-19.

#### H3

People’s perceived exposure to COVID-19 transmission can affect health protective behaviours against COVID-19 via their risk perception towards COVID-19.

#### H4

People’s perceived susceptibility to COVID-19 can affect health protective behaviours against COVID-19 via their risk perception towards COVID-19.

## Methods

### Study design and study area

**Fig. 2:**
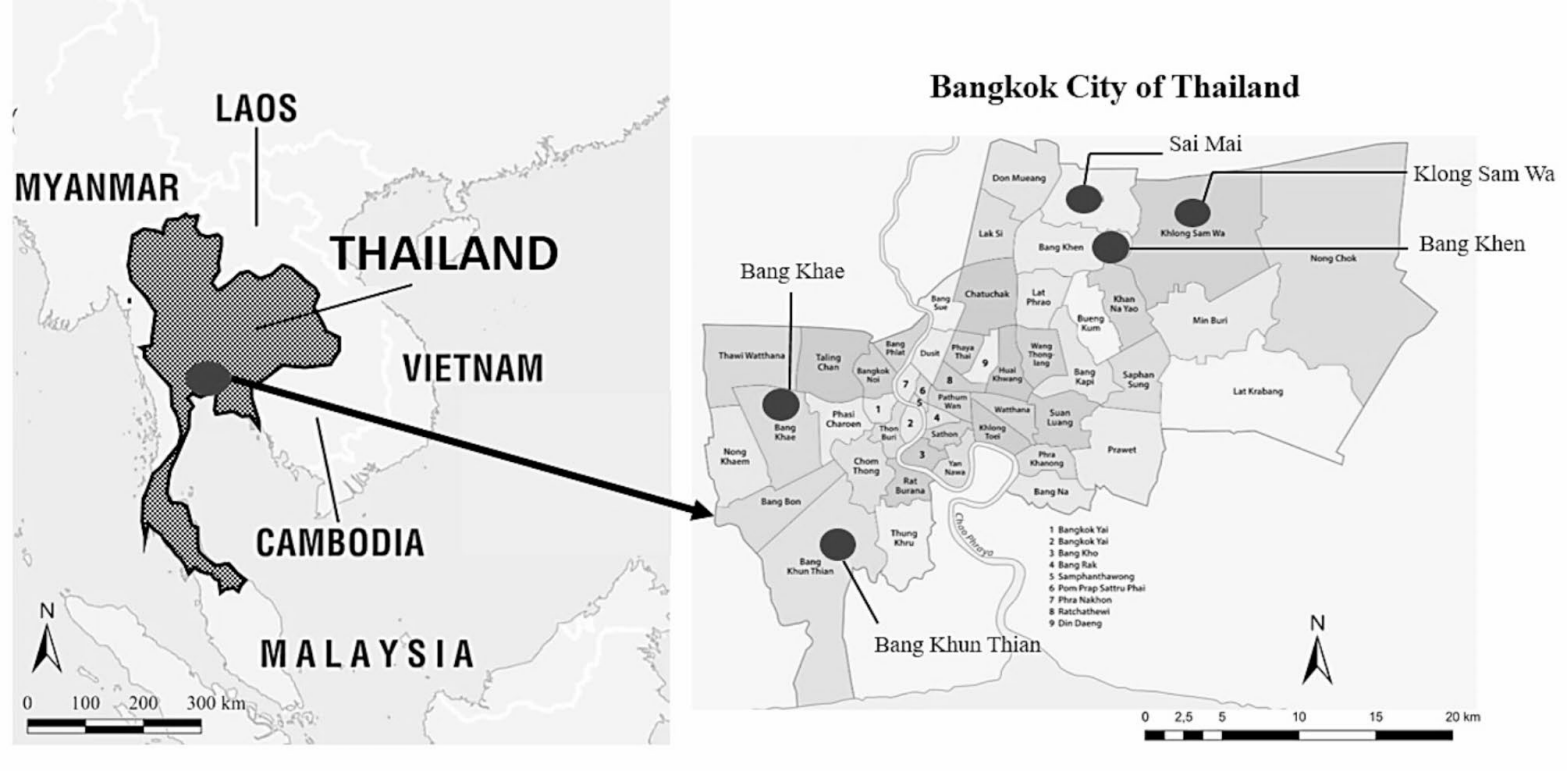
Study area

This study adopted a cross-sectional study design by using questionnaire surveys. Both online and face-to-face questionnaire surveys were conducted from 15 October to 9 November 2022 in Bangkok city of Thailand. Bangkok, the capital of Thailand, forms the country’s highest populated area, with approximately 5.5 million people living in an area of 1,568 km^2^ [85]. Bangkok contains 50 administrative districts. For this study, 5 districts with the largest population as of 2021 [86] were selected for questionnaire surveys (see Fig. 2). Those districts include Sai Mai having a population of 206,831 people, Khlong Sam Wa having a population of 206,437 people, Bang Khae having a population of 192,431 people, Bang Khen having a population of 186,200 people and Bang Khun Thian having 184,944 people.

### Participants and data collection

The population of this research is people in Bangkok city of Thailand. The sample size was calculated based on the formula of Cochran et al. [87]. The confidence level was set at 95%, and margin of error was set at 5%. A proportion of people who perform health protective behaviors against COVID-19 was set at 0.5 for calculating the max sample size (n). Accordingly, the result showed the appropriate sample size of 385 participants. However, to enhance reliability of data analysis, and to avoid insufficient datasets caused by a great number of incomplete survey responses and low response rate, this research recruited more participants. The estimated response rate was set as 70%, thus, approximately 550 participants were recommended. In total, questionnaire sheets were distributed to 550 research participants with a convenient sampling technique during the period of 15 October − 9 November 2022.

The questionnaire surveys were conducted in 5 selected administrative districts of Bangkok city. Approximately 110 residents in each target district were invited to participate in the data collection, and both face-to-face (F2F) and online questionnaire surveys were employed to collect data. The inclusion criteria were such as aged over 18 years, Thai citizens, and living in the survey area for more than 6 months. The exclusion criteria were healthcare workers such as medical staff and nurses; those who were having COVID-19 at the time of survey; or those who were caring for COVID-19-infected people at the time of survey. Because these groups of people were basically required to practice health protective measures against COVID-19. After the data collection, due to some incompletely responded questionnaire sheets, 29 samples were excluded, and 521 samples were suitable for data analysis.

### Ethical consideration

Before participants were requested to complete a questionnaire, the participants’ consent was received, and they were informed that their participation in the data collection was voluntary and had no negative impacts. In addition, participants were informed that they could deny answering sensitive questions (i.e., income, gender), if they feel discomfort. Ethical consideration for this research was evaluated and approved by the ethical research committee of King Mongkut’s University of Technology Thonburi (KMUTT). The date of approval is October 4th, 2022, and the approval number is KMUTT-IRB 2022/0928/252.

### Research tool

To collect the data, a questionnaire was developed based on a review of relevant literature. The questionnaire items used for measuring studied variables are presented in Table 1. The structure of questionnaire, explanation of the studied variables, types of questions and scales for the survey, are described in Table 1. The questionnaire’s validity was first evaluated by three experts. The Item-Objective Congruence (IOC) method was used to test content validity, and the questions having an IOC score lower than 0.50 were revised based on experts’ suggestion [88]. A pilot study was then conducted with 30 people to test the questionnaire’s reliability, which showed an acceptable Cronbach’s alpha (α) value of 0.87, exceeding the minimum requirement of 0.70 [89]. In addition, the scales for measuring risk perception towards COVID-19 (e.g., perceived probability of being infected by COVID-19 and perceived harmfulness of consequences caused by COVID-19 infection), and individuals’ understanding and perceptions of risk characteristics (e.g., understanding of threat severity, understanding of threat occurrence, perceived individual exposure, and perceived individual susceptibility) showed acceptable reliability with Cronbach’s alpha (α) values ranging from 0.74 to 0.89. All validated questionnaire items are shown in Table 1.

**Table 1:** Questionnaire items

Variable	Explanation	Items	Questions	Response Category
Health Protective Behaviours				
Health protective behaviours (Developed from the WHO’s recommendations for COVID-19 prevention and control measures) [9]	-Amount of medical face masks and antigen test kits used in a week -Individual’s participation in double mask wearing	Y1	How often do you undergo COVID-19 antigen testing in a week?	1 = rarely (less than 1 time/month) 2 = occasionally (1–2 times/ month) 3 = sometimes (3–4 times/ month) 4 = frequently (2–3 times/ week) 5 = often (more than 3 times/week)
		Y2	How many medical face masks do you use in a week?	1 = fewer than 5 masks/week 2 = 5–7 masks/week 3 = 8–10 masks/week 4 = 11–13 masks/week 5 = more than 13 masks/week
		Y3	How often do you put on double medical masks?	1 = rarely 5 = often
Health Risk Perceptions towards COVID-19				
Perceived probability of being infected by COVID-19 (PP) (Adapted from Shahnazi et al. [90] and Costa [91])	Individual’s belief regarding the chances of contracting COVID-19	PP1	How likely are you to be infected with COVID-19?	1 = no possibility 5 = high possibility
		PP2	How likely do you think that people you care about (such as family members) are to be infected with COVID-19?	
		PP3	For people who were previously infected by COVID-19, it is likely that they will be infected again?	
Perceived harmfulness of consequences caused by COVID-19 infection (PH) (Adapted from Magallares, [92])	Individual’s belief of how serious the virus is alongside the probable consequences of being infected	PH1	If I am infected with COVID-19, I might be sick for a long period.	1 = completely disagree 5 = completely agree
		PH2	If I am infected with COVID-19, my health might be impaired in the long run.	
		PH3	If I am infected with COVID-19, I might become severely ill.	
		PH4	If I am infected with COVID-19, the people I care about might become severely ill.	
		PH5	COVID-19 is dangerous to my health.	
		PH6	If I am infected with COVID-19, I might face financial problems.	
		PH7	If I am infected with COVID-19, my job or work tasks will be interrupted.	
Understanding and Perceptions of Risk Characteristics				
Understanding of threat occurrence (COVID-19 spreading; T) (Developed from Cardona et al.’s concept of hazard [25])	Individual’s understanding of a COVID-19 outbreak’s occurrence	T1	COVID-19 can spread easily.	1 = completely disagree 5 = completely agree
		T2	The COVID-19 outbreak will continue to last for a long time.	
		T3	COVID-19 can spread in any place.	
Understanding of threat severity (COVID-19; S) (Developed from Cardona et al.’s concept of hazard [25])	Individual’s understanding of COVID-19 dangers	S1	COVID-19 can cause deaths.	1 = completely disagree 5 = completely agree
		S2	The COVID-19 outbreak has interrupted future generations’ living.	
		S3	COVID-19 is harmful to human health.	
		S4	COVID-19 are still dangerous to human health, and I am concerned.	
Perceived exposure to COVID-19 (PE) (Developed from UNISDR’s concept of exposure [28])	Individual’s perception of their living conditions or daily activities, and whether they can easily cause COVID-19 infection	E1	My daily activities expose me to COVID-19.	1 = completely disagree 5 = completely agree
		E2	The people around me conduct daily activities that might cause me to contract COVID-19.	
		E3	Every day, I have to be in an environment that might cause me to contract COVID-19.	
Perceived susceptibility to COVID-19 (PSC) (Developed from UNDRO’s concept of susceptibility [27])	Individual’s perception of their health condition, and whether it is sensitive to COVID-19	SC1	I currently have a disease which might cause me to develop severe health issues upon COVID-19 infection.	1 = completely disagree 5 = completely agree
		SC2	My health condition is susceptible to the impacts of COVID-19.	

### Data analysis

Before the data analysis, all collected data were screened for completion, with any questionnaire sheets that were not completed excluded. The data analysis itself was divided into three steps. First, analyses of each variable’s descriptive statistics were performed. Then, a measurement model was estimated to test whether the questionnaire items had internal consistency when measuring each variable, as well as the scales’ construct and discriminant validity [93]. In the assessment of measurement model, the confirmatory factor analysis (CFA) was conducted to verify the construct validity of the scales used for measuring latent constructs [94]. The validity of measurement model was confirmed by fit indices (e.g., Root Mean Square Error of Approximation (RMSEA), Goodness of Fit Index (GFI), and Comparative Fit Index (CFI), and Chi-square(χ^2^)) [95, 96]. Based on this step, some questionnaire items with a low factor loading (< 0.60) were removed to enhance the internal consistency of each construct [97]. Additionally, to verify the measurement reliability and validity of each latent construct, Cronbach’s alpha (α) coefficients, average variance extracted (AVE) and combined reliability (CR) were calculated. Finally, the relationships outlined in the structural model were assessed by analysing the structural equal model (SEM) using IBM AMOS 2.5 and IBM SPSS statistics 22. The proposed relationship among the variables influencing health protective behaviours against COVID-19 is shown in Fig. 1. The model fit was tested via the chi-squared test (χ^2^), root mean square error of approximation (RMSEA), comparative fit index (CFI) and goodness of fit indices (GFIs) [96, 97]. Lastly, the risk characteristic constructs’ ability to predict infectious waste generation behaviours was derived.

## Results

### Characteristics of participants

The participant characteristics are shown in the Table 2. The proportion of female participants was 61.4% (*n* = 320) of the sample, while the proportion of male participants was 34.7% (*n* = 181). Approximately 3.8% of the sample did not want to identify their gender. Regarding age, most participants (*n* = 208, 39.9%) had the age between 20 and 30 years old. The participants younger than 20 years old was the minority (*n* = 21, 4.0%). The proportion of participants who had a bachelor’s degree was the majority (*n* = 272, 52.21%), while r participants who had an education level below a bachelor’s degree accounted for 12.48% (*n* = 65). Considering an average income, the proportion of participants who had an income lower than 15,000 baht or 420 USD was almost equivalent to the proportion of participants with an income more than 35,000 baht or 975 USD, 37% and 37.6% respectively. Additionally, most participants (*n* = 313, 60.1%) lived in their house, and approximately 58.4% (*n* = 304) had 1–3 family members.

**Table 2:** Demographic characteristics of participants (*n* = 521)

Demographic Characteristics		Number	Percentage (%)	
Gender	Male	181	34.7	
	Female	320	61.4	
	Do not want to identify	20	3.9	
Age (Years old)	Less than 20 years old	21	4.0	
	20–30	208	39.9	
	31–40	65	12.5	
	41–50	82	15.7	
	51–60	79	15.2	
	Higher than 60	66	12.7	
Education level	Lower than bachelor’s degree	65	12.5	
	Bachelor’s degree	272	52.2	
	Master’s degree	132	25.3	
	Doctoral degree	52	10.0	
Income (Baht)	Lower than 15,000 baht (420 USD)	193	37.0	
	15,000–35,000 baht (421–975 USD)	132	25.4	
	More than 35,000 baht (975 USD)	196	37.6	
Accommodation	Dormitory/Apartment	127	24.4	
	House	313	60.0	
	Rental house	39	7.5	
	Condominium	42	8.1	
Family members	1–3 persons	304	58.4	
	4–6 persons	197	37.8	
	More than 6 persons	20	3.8	

### Health protective behaviours

The survey results revealed that most of the research participants (38%) tested for COVID-19 infection by using ATKs approximately 1–2 times per month. Participants who tested for COVID-19 less than 1 time per month accounted for 31% of the study population. Approximately 2% reported using ATKs more than 3 times per week. Regarding the number of face masks used to prevent COVID-19, the results showed that most participants (52.2%) used 5–7 masks per week. Approximately 20% reported using 8–10 face masks per week, and about 7% reported using more than 13 per week. Regarding the use of double masks for COVID-19 prevention, the results revealed that most participants (approximately 38%) reported sometimes wearing double masks, while approximately 28% reported never wearing double masks. Participants who reported regularly wearing double masks accounted for 8.6% (see Table 3).

**Table 3:** Health protective behaviours (*n* = 521)

Health Protective Behaviours		*n*	%
Frequency of COVID-19 antigen test kit use (ATK)	Less than 1 time/month	165	31.7
	1–2 times/month	176	33.8
	3–4 times/month	145	27.8
	2–3 times/week	24	4.6
	More than 3 times/week	11	2.1
Quantity of medical face masks used in a week (MM)	Fewer than 5 masks/week	95	18.2
	5–7 /masks/week	272	52.2
	8–10 masks/week	102	19.6
	11–13 masks/week	16	3.1
	More than 13 masks/week	36	6.9
Frequency of wearing double medical masks (DMM)	Never	148	28.4
	Rarely	96	18.4
	Sometimes	199	38.2
	Often	33	6.3
	Regularly	45	8.7

### Descriptive statistics and measurement Model

#### Health risk perceptions

After screening the data for completion, mean and standard deviation were calculated of study variables (see Table 4). A second order confirmatory factor analysis (CFA) was also performed to test the interactions between each health risk perception construct and its observed indicators. It was assumed that health risk perception was a general latent variable which could in turn be explained by two first-order factors, specifically perceived probability of contracting COVID-19 (PP) and perceived harmfulness of the virus’s impacts (PH). After excluding three observed indicators of PH (PH5–7) with low loading estimates (< 0.05) [87, 88], the model was determined to have an acceptable fit with the data (Brown [98]; chi-square (χ^2^) = 6.815; degree of freedom (df) = 6; *p* = 0.338; ratio of chi-square/degree of freedom (χ^2^/df) = 1.136; GFI = 0.996; Tucker–Lewis Index (TLI) = 0.998; CFI = 1.000; AGFI = 0.983; RMSEA = 0.016). The two latent variables of the second-order CFA model explained the second-order latent “health risk perception” variable. The standardized beta coefficients obtained from the PP and PH latent variables were β = 0.799, *p* < 0.01 and β = 0.75, *p* < 0.01, respectively. Based on these results, an indicator of health risk perception was created by calculating an average score from the 7 items in the two first-order latent variables (M = 3.187, SD = 0.750).

**Table 4:** Confirmatory factor analysis results for the constructs of health risk perception (*n* = 521)

Variable	Items	Mean	SD	Factor Loadings (> 0.6)	CR (> 0.7)	AVE (≥ 0.5)	Cronbach’s Alpha (> 0.7)
Perceived probability of being infected by COVID-19 (PP)	PP1	2.889	0.852	0.888	0.855	0.664	0.836
	PP2	2.962	0.883	0.838			
	PP3	3.155	1.110	0.708			
Perceived harmfulness of consequences caused by COVID-19 infection (PH)	PH1	3.378	1.108	0.778	0.822	0.536	0.837
	PH2	3.607	1.140	0.688			
	PH3	3.232	1.143	0.720			
	PH4	3.274	1.155	0.739			
	PH5	Deleted					
	PH6	Deleted					
	PH7	Deleted					
Risk perception (Second-order factor)	PP	3.002	0.829	0.799	0.723	0.568	0.720
	PH	3.373	0.931	0.705			
	Average	3.187	0.750				

Note. SD denotes the standard deviation. AVE denotes Average Variance Extracted and CR denotes Construct Reliability

Considering the loadings of the observed indicators shown in Table 4, the loadings were acceptable, as all items were significantly loaded on their designated latent variables (*p* < 0.001), and had a standardized factor loading > 0.60 that indicated convergent validity [99]. Two latent variables (PP and PH) were also significantly loaded on health risk perception. Moreover, the reliability and validity of the latent variables was examined via three indicators: composite reliability (CR), average variance extracted (AVE) and Cronbach’s α. AVE reflects the average amount of variance that a construct can explain in its indicators, with an AVE of ≥ 0.5 indicating suitable convergent validity [99]. This study’s data yielded AVE scores of 0.536 and 0.644, greater than the generally accepted minimum of 0.5 [100]. CR, meanwhile, is calculated to test the reliability of a latent variable [101], and implies how each indicator is consistent in what it intends to measure. The model assessment showed CR values of 0.822 and 0.855, which were greater than the acceptable threshold of 0.70, indicating that the latent variable measurement model had good reliability [100]. For Cronbach’s α, which is calculated to assess the internal reliability of the given measures, the values of the two latent variables were greater than the threshold of 0.70, indicating internal reliability [100]. Based on the calculations of these three indicators, the model was internally consistent, while the observed indicators substantially measured the constructs of health risk perception.

#### Determinates of health risk perception

CFA was performed to test the measurement reliability and validity of the factors that potentially affect health risk perception. CFA indicates the factor loading for each item in each variable. This study had four variables that were assumed to have an effect on health risk perception: understanding of threat occurrence (T), understanding of threat severity (S), perceived exposure (PE) and perceived susceptibility to COVID-19 (PSC). Statistical analysis again indicated the model’s acceptable fit with the data (Brown, [98]; χ^2^ = 36.373; df = 34; *p* = 0.359; χ^2^/df = 1.170; GFI = 0.982; TLI = 0.996; CFI = 0.998; AGFI = 0.963; RMSEA = 0.018). As seen in Table 5, the factor loadings of all items were above the standard value of 0.60 [99], indicating convergent validity. To verify the convergent validity of the model’s latent variables, AVE and CR were also calculated, yielding AVE values ranging from 0.640 to 0.779, which were greater than acceptable minimum of 0.5 [100], and CR values ranging from 0.842 to 0.891, which also met the acceptable threshold of 0.7 [100]. In addition, Cronbach’s α was calculated to evaluate the measures’ internal reliability. The Cronbach’s α coefficients for the scales ranged from 0.864 to 0.892, which were above the threshold of 0.7 [100]. The measurement model was thus internally consistent, and all items could be used to measure factors that affect health risk perception.

**Table 5:** Confirmatory factor analysis results for determinants of health risk perceptions (*n* = 521)

Variable	Items	Mean	SD	Factor Loadings (> 0.6)	CR (> 0.7)	AVE (≥ 0.5)	Cronbach’s Alpha (> 0.7)
X1: Understanding of threat occurrence (T)	T1	4.100	0.991	0.882	0.891	0.732	0.892
	T2	4.054	0.985	0.868			
	T3	4.355	0.932	0.815			
X2: Understanding of threat severity (S)	S1	3.225	1.169	0.759	0.890	0.670	0.878
	S2	3.835	1.137	0.828			
	S3	3.806	1.130	0.867			
	S4	3.566	1.239	0.817			
X3: Perceived exposure to COVID-19 (PE)	E1	3.280	1.164	0.785	0.842	0.640	0.864
	E2	3.322	1.168	0.857			
	E3	3.029	1.190	0.754			
X4: Perceived susceptibility to COVID-19 (PSC)	SC1	2.714	1.420	0.87	0.876	0.779	0.875
	SC2	2.862	1.226	0.895			

Note. SD denotes the standard deviation. AVE denotes Average Variance Extracted and CR denotes Construct Reliability

Additionally, the correlation analyses were performed to verify discriminant validity. According to Pearson’s correlation analysis, the correlations between the study variables (T, S, PE, PSC, PP, PH, ATK, MM and DMM) were statistically significant (*p* < 0.05; Fornell & Larcker, [100]), confirming discriminant validity. The coefficient values were not greater than 0.60, indicating that there was no problem with multicollinearity [102]. In this way, the structural equation model (SEM) analysis could be carried out to test the developed conceptual model presented in Fig. 1.

### Structural model assessment

**Fig. 3:**
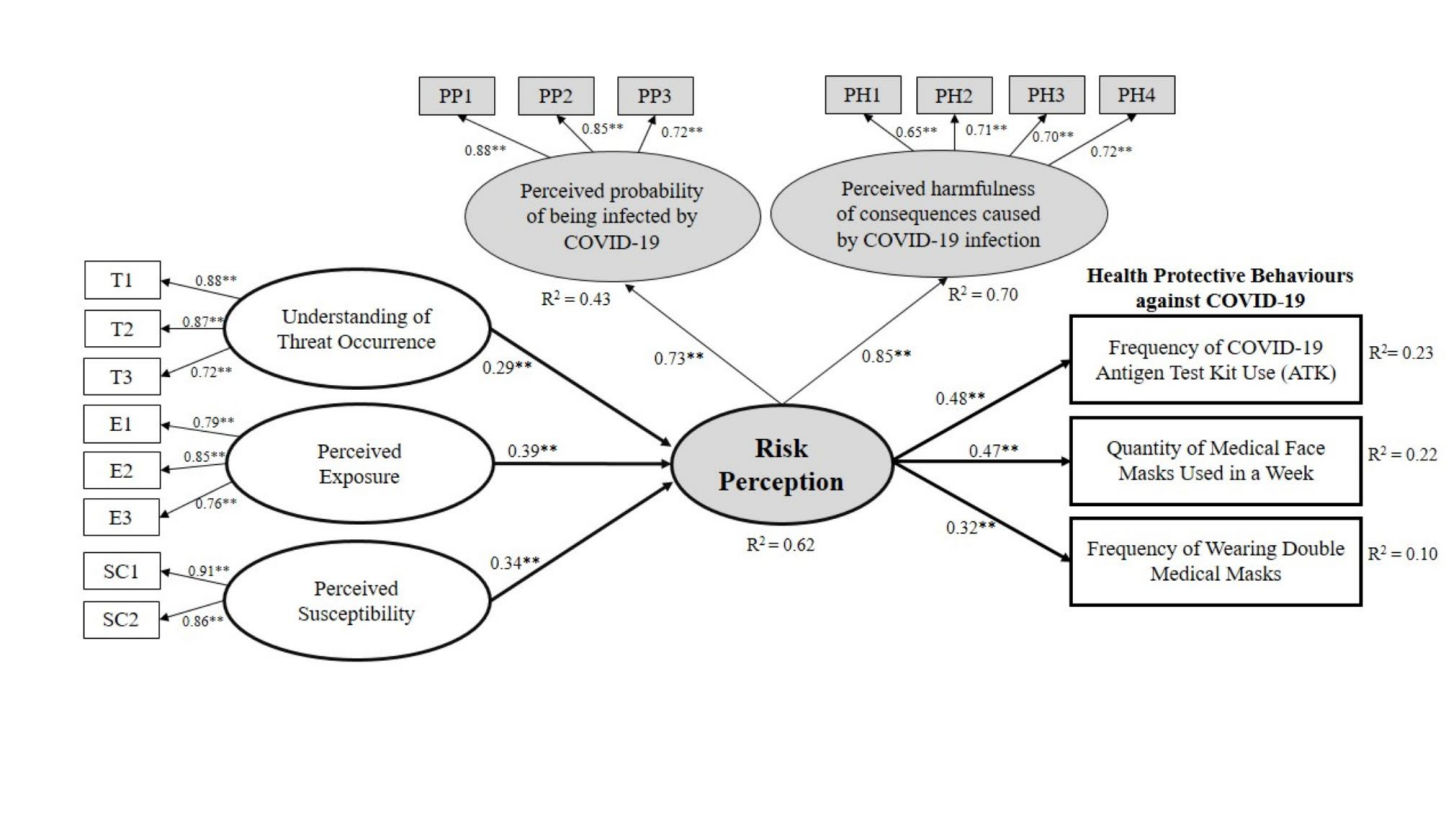
The study’s structural equation modelling (SEM; ** *p* < 0.001)

A SEM analysis was performed to test the association among study variables, that is, risk characteristic constructs, health risk perception and health protective behaviours. The study first checked the overall fit of the model with the data, with the results indicating that the model did not fit with the observed data, and the latent variable “S” (understanding of threat severity) did not significantly affect health risk perception. Therefore, to improve the model’s fit, “S” was removed. The proposed model then fit perfectly with the data, as the χ^2^ value was not statistically significant (χ^2^ = 105.166; df = 97; *p* = 0.268), and χ2/df was 1.084, which is not greater than 5.0 [99]. Other statistical indices also implied the acceptance of the structural model. The GFI value was 0.979, which was greater than 0.90, indicating a close fit between the observed data and the structural model [99]. The RMSEA value was 0.013, less than 0.08 and thus indicating a reasonable approximation of the data [103]. The CFI value was then calculated to explain the discrepancy function adjusted for sample size; this value was acceptable at 0.998, which is greater than 0.90 [104]. The analysis also yielded an incremental fit index (IFI) value of 0.998, which is greater than 0.900 and thus indicates the proposed model’s acceptability [105]. The normed fit index (NFI) and TLI values also met the standard value of 0.9, exhibiting that the structural model perfectly fit the observed data [105]. Overall, the proposed structural model was statistically acceptable (see Fig. 3).

### Effect of risk characteristic constructs on health risk perception, and the effect of health risk perception on health protective behaviours

The path coefficients among the study variables were examined next. The hypothesized paths from risk characteristic constructs (e.g., understanding of threat occurrence (T), perceived exposure (PE) and perceived susceptibility to COVID-19 (PSC)) to the latent variable of risk perception were statistically significant. Namely, T (β = 0.292; t = 5.289; *p* < 0.001), PE (β = 0.388; t = 5.525; *p* < 0.001) and PSC (β = 0.336; t = 3.727; *p* < 0.001) significantly affected risk perception. In this way, H2 was rejected, as it was excluded from the model.

Considering standardized beta values, perceived exposure to COVID-19 transmission (PE) had the greatest impact on risk perception, and understanding of threat occurrence (T) had the lowest impact. The predicted paths from risk perception also significantly affected three types of health protective behaviours: frequency of COVID-19 ATK use (β = 0.478; t = 8.467; *p* < 0.001), quantity of medical face masks used in a week (β = 0.465; t = 8.313; *p* < 0.001) and frequency of wearing double medical face masks (β = 0.320; t = 5.833; *p* < 0.001). When considering the standardized beta values, the impact of health risk perception on frequency of COVID-19 ATK use (ATK) was the greatest (see Table 6).

**Table 6:** Path coefficient estimate of the revised model

Paths	Estimate	S.E.	C.*R*.	β
T → Risk perception	0.183	0.035	5.289	0.292**
PE → Risk perception	0.23	0.042	5.525	0.388**
PSC → Risk perception	0.144	0.039	3.727	0.336**
Risk perception → ATK	0.856	0.101	8.467	0.478**
Risk perception → MM	0.872	0.105	8.313	0.465**
Risk perception → DMM	0.718	0.123	5.833	0.320**

Note. ** *p*-value < 0.001, S.E. denotes the standard error. C.R. denotes Construct Reliability, and β denotes the standardized regression coefficient

### Mediation effect of risk perception on the relationship between risk characteristic constructs and health protective behaviours

To test the mediating effect of risk perception on the relationship between risk characteristic constructs and health protective behaviours, bootstrapping analysis was performed. The results revealed that risk perception mediated the effect of risk characteristic constructs T, PE and PSC on each type of health protective behaviour (see Table 7). Namely, understanding of threat occurrence (T), perceived exposure to COVID-19 transmission (PE) and perceived susceptibility to COVID-19 (PSC) had a significant indirect effect on health protective behaviours via risk perception. In this way, H1, H3 and H4 were accepted. The study then compared the indirect effects of T on each type of health protective behaviour. The bootstrapping analysis revealed that the indirect effect of T on frequency of COVID-19 ATK use (ATK) via risk perception was the greatest (0.205), and its indirect effect on the quantity of medical face masks used in a week (MM) was the lowest (0.185). For perceived exposure, the indirect effect of PE on the frequency of wearing double medical face masks (DMM) was the greatest (0.194), whereas its indirect effect on MM was the lowest (0.163). Perceived susceptibility to COVID-19 (PSC) had the greatest indirect effect on ATK (0.105) and the lowest indirect effect on DMM (0.095).

**Table 7:** Mediation test using bootstrapping. (standardized indirect effect) (*n* = 521)

Paths	Bootstrapping		Confidence Interval 95% Bias-Corrected CI		Statistically significant
	IE	Boot S.E.	Boot LLCI	Boot ULCI	
**Indirect effect of understanding of threat occurrence (T)**					
T → Risk perception → ATK	0.205	0.031	0.145	0.269	Yes*
T → Risk perception → MM	0.185	0.030	0.129	0.248	yes*
T → Risk perception → DMM	0.186	0.031	0.127	0.247	yes*
**Indirect effect of perceived exposure (PE)**					
PE → Risk perception → ATK	0.191	0.030	0.135	0.250	yes*
PE → Risk perception → MM	0.163	0.027	0.112	0.219	yes*
PE → Risk perception → DMM	0.194	0.033	0.134	0.262	yes*
**Indirect effect of perceived susceptibility (PSC)**					
PSC → Risk perception → ATK	0.105	0.018	0.071	0.142	yes*
PSC → Risk perception → MM	0.101	0.018	0.068	0.1372	yes*
PSC → Risk perception → DMM	0.095	0.019	0.061	0.134	yes*

Note. IE denotes the effect explained by mediators, Boot S.E. denotes bootstrap standard errors, Boot LLCI and ULCI denote the lower and upper limits of the CI, respectively., *Given 0 does not fall within the confidence interval (Boot LLCI and Boot ULCI)

## Discussion

The COVID-19 situation is changing over time. This change could affect the way people perceive the risks associated with COVID-19, thus affecting their health protective behaviours (e.g. face mask wearing and ATK testing). Practising health protective behaviours to prevent and control COVID-19 consequently contributes to good health outcomes. This study was based on the assumption that how people perceive risks affects their health protective behaviours, and how people’s understanding and perceptions of risk characteristics related to COVID-19 could shape the way people construct their health risk perception. These characteristics are hazard (threat occurrence and threat severity), individual susceptibility and exposure to the virus.

This study first revealed that individuals’ health risk perceptions were significantly determined, in order, by their perceived exposure to the virus, perceived susceptibility and understanding of the possibility of threat occurrence. In contrast, understanding of threat severity was not a significant predictor of health risk perception. The combination of individuals’ understanding and perceptions of these three risk characteristics could explain 62% of variance in overall health risk perceptions. This finding supports the notion of Slovic et al. [106], who proposed that individuals’ risk perception is constructed based on their understanding of risk characteristics and affective responses (e.g. dread, worry) to a particular health threat. However, in assessing all risk characteristics, this study demonstrated that individuals’ understanding of a threat severity (COVID-19) is not a significant factor that affects individuals’ risk perception. This implies that though the virus itself is perceived as less dangerous due to people’s increased self-immunity (i.e. vaccination), individuals can still construct health risks to such a degree that they perceive the virus as harmful to human health. This is because individuals evaluate the degree of facing health risks mainly based on their perceived exposure (e.g. being in a crowded environment, travelling, living with people who always do outside activities) and individual susceptibility (e.g. poor health conditions or having a chronic disease). The results of this study thus contradict many previous studies that have reported the significant effect of perceived severity of a threat (or its catastrophic consequences) on individuals’ health risk perception [107, 108].

This study does, however, strengthen the psychometric paradigm proposed by Slovic [22] by providing evidence that individuals’ perceived control over their exposure plays an important role in shaping risk perceptions of COVID-19. Feeling lack of control over their exposure can cause fear in individuals, thus leading to the development of a greater risk perception. In turn, individuals’ perceived catastrophic consequences of a threat might not be important, particularly in situations where people are familiar with the threat. As this study was conducted from October to November 2022, when the COVID-19 outbreak had been present for almost 3 years, the participants were quite familiar with the virus.

Individuals’ perceived susceptibility or weakness to a health threat was found to be a significant determinant of COVID-19 risk perception. Namely, the participants who had a higher perception of their own weakness to COVID-19 (e.g. having a chronic disease) tended to construct a higher health risk perception. Many studies have also reported the significant effect of individuals’ perceived susceptibility to a disease on health risk perception [22, 48, 104]. People with vulnerable conditions might feel that they are sensitive to a disease threat; consequently, they construct a feeling of fear or worry that leads to health risk perception [22]. For instance, the study of Adachi, et al. [48] revealed that participants who reported poorer health conditions were more likely to report a significant higher level of risk perception towards COVID-19. Furthermore, this study showed that individuals’ understanding of threat occurrence (existence or occurrence of COVID-19 spreading) significantly influenced a degree of health risk perception in the participants. However, its power to predict health risk perception was weaker than perceived exposure and perceived susceptibility. This is attributable to people still needing to know about a hazard’s possibility of occurrence to initiate evaluations of their risk, and thus decide whether to take preventive measures.

Additionally, this study revealed that health risk perception mediated the effect of individuals’ perceptions of risk characteristics on health protective behaviours. Health risk perception significantly directly affected health preventive behaviours. The direct effect of risk perception on health protective behaviours can be supported by the HBM [52], as well as many previous studies that confirm that health risk perception contributes to the practice of health protective measures [109–111]. For instance, Bruine de Bruin and Bennett [19] found that people were more likely to comply with health protective measures if they had a high level of perceived risk related to COVID-19, as based on their perceived possibility of infection and infection fatality. Similarly, Tang and Wong [112] reported that health risk perception based on the perceived probability of being infected or the perceived harmfulness of illness among adult Chinese individuals in Hong Kong encouraged them to comply with health-related guidelines. Leppin and Aro [113] also found that risk perception only predicts individuals’ protective behaviours when people possess self-efficacy or response efficacy.

## Recommendations and conclusion

This study can provide practical implications for the development of communication strategies which can motivate people to participate in health protective behaviours against COVID-19. Even though, the findings may need further explorations to be generalized to the public at large due to the limitations related to the sample size and unique characteristics of the samples who were urban populations in Bangkok city of Thailand, the results could provide evident-based risk communication efforts based on the results generated from the scientific and analytical method. Risk communicators (e.g., health professionals, healthcare staff, community leaders, and the government) could gain the deep understanding of how people constructed the risk perception of COVID-19 infection and illness during the post-pandemic era, and how the risk perception could influence the performance of health protective behaviours against COVID-19. Types of risk messages which can enhance or reduce people’s risk perception are identified.

The study revealed that perceived exposure had the strongest impact on individuals’ risk perception, and risk perception in turn significantly affected all three types of health protective behaviours (frequency of COVID-19 ATK use, quantity of medical face masks used in a week and frequency of wearing double medical face masks). In addition, the study revealed that people’s risk perception was constructed based on their perceived susceptibility (i.e., poor health conditions, having chronic disease) and their understanding of threat occurrence. Thus, vulnerable groups, such as people with chronic diseases or poor health conditions, are likely to be active to act against COVID-19. Based on the current COVID-19 situation, even though, the pandemic is better than before, and the virus is perceived as less harmful to human health, people can construct risk perceptions as long as COVID-19 endures and people feel susceptible to the virus. These constructed risk perceptions are essential in promoting the performance of health protective behaviours against COVID-19.

To promote the construction of risk perception towards COVID-19, communicating the public with these three types of risk message can be effective. The first type of risk message is information related threat occurrence such as the possibility of virus transmission, characteristics of transmission, possibility of virus mutations and the exist of COVID-19 pandemic. If people perceive that the pandemic still exists, and the virus is contagious, people will start to think about their possibility to be inflected. The second type of risk message is information about exposure to COVID-19 such as the risk of virus transmission in particular environments (i.e., crowed and narrow places and areas with poor ventilation) and in particular groups of people (i.e., careless people and people having a social lifestyle associated with many people such as parties, events, and travel). If people could understand COVID-19 exposure conditions, they could judge their possibility to be infected with the virus based on their living contexts and lifestyles. The third type of risk message is information about vulnerable conditions to COVID-19 (e.g., elderly people and people with chronic diseases such as hypertension, cardiovascular diseases, diabetes, obesity, and cancer). This type of information can help people judge the seriousness of illness if they are infected. If the health impacts caused by infection are perceived high, people are likely to construct a high risk perception. The result of this study showed that three types of perceptions and understanding of these risk characteristics could explain 62% of variances in health risk perception which in turn influenced health protective behaviours. Furthermore, it should be highlighted that during the post-pandemic era, communicating with the public about severity of COVID-19 inflections (e.g., number of deaths, mortality rate, possible severe symptoms) as always performed during the pandemic period, might not be successful in promoting self-protective behaviours. Because people become familiar with the nature of virus transmission and severity of infections. This finding could provide the theoretical perspective on health risk perception. Namely, when people are familiar with a threat, people’s understanding and perceptions of threat severity (e.g., the severity of symptoms caused by the virus, the probability of death) might not be influential to the construction of health risk perception.

## Study limitations

First of all, it is important to note that the findings of this research are based on self-reported data from a specific urban population (Bangkok city of Thailand). Thus, the study has limited capacity to generalize the results to the populations at large. Second, this study contains some limitations related to uncontrolled or unmeasured variables that could have influenced the results of the study. Those uncontrolled variables are such as socio-demographic factors, social influence, and governmental policies. Third, health risk perception, together with its significant determinants, predicted 23% of the variance in the frequency of COVID-19 ATK use, 22% of the variance in quantity of medical face masks used in a week but only 10% of the variance in frequency of wearing double medical face masks. The leftover total variance might be explained by social factors (i.e., peer influence, social norms), the impact of governmental policies, perceived self-efficacy and response efficacy, as recommended by Rogers’s [17] PMT and Leppin and Aro [113]. Further research may therefore include an efficacy variable, socio-demographic factors, and social influence to enhance the model’s ability to predict individuals’ health protective behaviours.

## Data Availability

The datasets used and analyzed during the current study are available from the corresponding author on reasonable request.
